# The joint Simon effect: a review and theoretical integration

**DOI:** 10.3389/fpsyg.2014.00974

**Published:** 2014-09-05

**Authors:** Thomas Dolk, Bernhard Hommel, Lorenza S. Colzato, Simone Schütz-Bosbach, Wolfgang Prinz, Roman Liepelt

**Affiliations:** ^1^Department of Psychology, Max-Planck-Institute for Human Cognitive and Brain SciencesLeipzig, Germany; ^2^Research Group: Heterogeneity and Inclusion, Faculty of Human Science, University of PotsdamPotsdam, Germany; ^3^Institute for Psychological Research and Leiden Institute for Brain and Cognition, Leiden UniversityLeiden, Netherlands; ^4^Independent Research Group “Body and Self,” Max-Planck-Institute for Human Cognitive and Brain SciencesLeipzig, Germany; ^5^Institute for Psychology, University of MuensterMuenster, Germany

**Keywords:** joint action, joint Simon effect, social cognition, stimulus-response compatibility, referential coding, review

## Abstract

The social or joint Simon effect has been developed to investigate how and to what extent people mentally represent their own and other persons' action/task and how these cognitive representations influence an individual's own behavior when interacting with another person. Here, we provide a review of the available evidence and theoretical frameworks. Based on this review, we suggest a comprehensive theory that integrates aspects of earlier approaches–the Referential Coding Account. This account provides an alternative to the social interpretation of the (joint) go-nogo Simon effect (aka the social Simon effect) and is able to integrate seemingly opposite findings on joint action.

## Introduction

Throughout life, people are constantly engaging in social interactions, be it while playing games, dancing, or working toward a common goal together. While doing so, they seem to share an implicit understanding of what *joint action* means, implies, and requires. The term is typically defined as the ability to coordinate one's own actions with those of others “in space and time to bring about a change in the environment” (Knoblich and Sebanz, 2006; p. 100).

Despite people's intuitive understanding of joint action, its underlying cognitive mechanism(s) are far from being fully understood, however. Scientific research over the last decades improved our understanding of how perception and action are linked (i.e., by sharing common representations; Hommel et al., 2001), how individuals select task-relevant information, predict upcoming actions, and integrate predicted effects of one's own and others' actions (Wilson and Knoblich, 2005). However, this research studied single individuals performing various cognitive and/or behavioral tasks in isolation. Since social beings spend most of their time interacting and communicating with others, a major function of human cognition is likely to facilitate joint action (Frith and Wolpert, 2004; Tomasello et al., 2005; Tomasello, 2009; Hasson et al., 2012). What is unclear, however, is whether processing information about other people and their activities requires special, dedicatedly “social” mechanisms, as some authors have claimed (Sebanz et al., 2006a; Sebanz and Knoblich, 2009), or whether universal information-processing mechanisms are sufficient (Hommel et al., 2009; Dolk et al., 2011, 2013a). For instance, while proponents of dedicated social mechanisms tend to take it as self-evident that synchronizing the behavior of multiple individuals requires the cognitive representation of the others' goals and actions, there are numerous examples of well-synchronized group behavior, such as “schooling” in fish swarms (Shaw, 1978), that are very unlikely to rely on such high level representations. Thus, one of the key questions of today's cognitive science is: how and to what extent do individuals mentally represent their own and others' actions, and how do these representations influence, shape, and constrain an individual's own behavior when interacting with others?

First experimental approaches targeting these issues compared performance on cognitive tasks that were either carried out alone (*single setting*) or together with another person (*joint setting*). Quite similar to tennis, say, individuals were thus responsible for the entire performance in the single setting but were taking turns in the joint setting, where they shared labor and responsibility with another person. The most prominent cognitive task that has been tested in single and joint settings is the *Simon task* (Simon, 1969). Interestingly, performing this **Stimulus-Response Compatibility (SRC)** task alone (*standard Simon task*) or when taking turns with another person (*joint/social go-nogo Simon task*) often leads to comparable performance, i.e., a Simon effect (Sebanz et al., 2003), which inspired the idea of human cognition to be mandatorily social (e.g., Tsai and Brass, 2007; Welsh, 2009; Müller et al., 2011a). However, not only has it remained unclear what that actually means but there is also still a considerable lack of understanding of the precise processes governing joint task performance, apart from the fact that more recent findings fail to provide support for a purely social picture of human cognition.

KEY CONCEPT 1. Stimulus-Response Compatibility (SRC) Reflects the amount of compatibility between given stimulus and corresponding response-features. High levels of SRC are commonly associated with shorter RTs as compared to longer RTs due to low levels of SRC. Beside the Simon effect, the Flanker, the Stroop, and the spatial-numerical association of response codes (SNARC) are classical SRC-paradigms.

In the following, we provide an overview of the basic method and the available findings in the domain of joint action (with a strong focus on the social Simon effect). We will then make an attempt to integrate the available evidence into a comprehensive theory–the **Referential Coding** Account. As we will point out, this theoretical account provides an alternative to the social interpretation of the (joint) go-nogo Simon effect and explains seemingly contradictory observations from go-nogo Simon task performance.

KEY CONCEPT 2. Referential Coding Is required to discriminate concurrently activated event-representations (e.g., because of endogenous preparation, stimulus-induced activation, and/or cross talk) that refer to conflicting (self- and/or other-generated) action alternatives. Although *location* is critical in Simon-tasks, other event-features are also likely to enable event discrimination in a given task context.

## The joint Simon paradigm

A tremendous amount of studies provide behavioral as well as neurophysiological evidence for the functional implications of **shared representations** between action and perception—within and between individuals (e.g., Adolphs, 2003; Wilson and Knoblich, 2005; Amodio and Frith, 2006). For instance, perceiving or imagining another person's actions activates one's own cognitive representations involved in planning and executing similar actions, which has been taken to provide a basic mechanism that enables individuals to identify ongoing actions and to anticipate upcoming action events. However, evidence for the functional equivalence between imagining, perceiving and executing an action (common coding; see Prinz, 1990, 1992, 1997; Hommel et al., 2001, for an extension known as the *Theory of Event Coding* [TEC]) has been obtained under conditions where participants either passively observed other individuals performing certain actions or where they had to imitate these actions (Brass and Heyes, 2005; Liepelt et al., 2008a, 2010). In contrast, when engaging in joint action, individuals are often required to perform complementary parts of a given task, i.e., taking turns rather than acting at the same time. How one's own action planning and execution is influenced by the presence of others, their task and their planned and executed actions during social interaction is just beginning to be understood. The most prominent paradigm that has been developed to address this issue is known as the *joint Simon paradigm*. In this paradigm two participants share a task that in the *standard* version of the Simon task is performed individually.

KEY CONCEPT 3. Shared representations As cognitive representations are intrinsically individual and private they can only arise and operate in individual minds. Consequentially, shared representations are cognitive representations of two or more individuals that refer to the same reference object/event, thereby being both: private - existing in individual minds - and shared - referring to the same reference.

### The standard Simon task

In the standard Simon task, participants are required to carry out spatially defined responses (e.g., left/right key presses) to non-spatial stimulus attributes (e.g., auditory pitch or visual color; Simon, 1969; Simon and Craft, 1970) that randomly appear on the left or right of the participant. For example, participants are instructed to press a right key whenever they perceive a high-pitched tone and a left key in response to a low-pitched tone. Even though stimulus location (left or right) is entirely task-irrelevant, responses are typically faster when they spatially correspond to the stimulus signaling them. That is, spatial *Stimulus-Response* (S-R) correspondence facilitates task performance, whereas non-corresponding S-R pairs commonly lead to impaired performance—a phenomenon that is known as the Simon effect (*SE*; Simon, 1990).

The *SE* has been replicated featuring diverse stimulus displays (e.g., auditory, somatosensory, and visual; see Proctor and Vu, 2006; Hommel, 2011 for reviews) and a variety of S-R arrangements (e.g., horizontal and vertical). Most models explain the SE by assuming that a match between spatial stimulus locations and spatial response locations (or features thereof) facilitates response selection, be it (1) because of a direct association between them (e.g., Kornblum et al., 1990; De Jong et al., 1994) or (2) because the identity of the codes representing these locations (e.g., Hommel, 1993; Hommel et al., 2001), or (3) because attentional shifts appearing in response to the lateralized stimulus presentation prime spatially corresponding action events (e.g., Nicoletti and Umiltà, 1989, 1994). A mismatch between stimulus and response locations in contrast is assumed to create competition between the primed response and the response required by the instruction (dual-route model; Kornblum et al., 1990).

According to this logic, the SE should not be obtained if there is no alternative response (location). Indeed, if the task is turned into a go-nogo task by having participants perform with only one response key, thus eliminating the spatial dimension of the responses (*individual* or *solo go-nogo Simon task*), the SE usually disappears (Hommel, 1996). And that makes sense: In the absence of a feature overlap between spatially varying stimuli and responses, S-R relations need no longer to be discriminated and are therefore no longer spatially coded (Liepelt et al., 2011; Hommel, 2013), eliminating the interference caused by competing response tendencies and thus, the *SE*.

### The joint Simon task

Interestingly, however, distributing the complementary go-nogo parts of the standard Simon task across two participants, so that each individual is responsible for operating one of the two buttons in response to their assigned stimulus (i.e., joint/social go-nogo Simon task), also lead to an SE (Sebanz et al., 2003)—a phenomenon known as the joint Simon Effect (JSE). That is, similar to the standard *SE*, observed when one participant is responsible for both responses, participants in the joint go-nogo Simon task respond faster if the assigned stimulus spatially corresponded to the actor's response key.

The finding of a JSE has been considered to demonstrate that participants share a complementary, pre-instructed task set and create not only a cognitive representation of their own action but also co-represent (at least) the action of their co-actor (Sebanz et al., 2003). By representing one's own and another person's actions, the spatial dimension of the responses is thought to be represented as well, thereby reintroducing a feature-overlap of spatial S-R dimensions (Sebanz et al., 2003). Like in the standard SE, a match between spatial stimulus locations and spatial response locations facilitates task performance, whereas a mismatch induces response interference (Sebanz et al., 2003; Ferraro et al., 2011). The JSE is thus attributed to *action co-representation*, which is assumed to be an automatic and dedicated “social” process (Knoblich and Sebanz, 2006; Sebanz et al., 2006a; Sebanz and Knoblich, 2009).

The finding of the JSE and the far-reaching conclusions it has been taken to suggest have ignited further behavioral, neuroscientific, and clinical research investigating the representations underlying the JSE. The following sections will provide a brief overview of these studies.

#### Behavioral findings

One of the questions that were tackled by behavioral studies was whether the S-R compatibility effects in the joint action condition depend on the spatial relation between (left and right) stimuli and response keys (*response-based* compatibility) or on the spatial relation between (left and right) stimuli and responding agents (*agent-based* compatibility). In contrast to the standard Simon task, in which a single participant is responding with the left and right hand, the joint Simon task requires participants to sit next to each other and to respond with just one hand—usually the right or dominant one. Hence, in the joint Simon task, the spatial origin of the agents' bodies and the spatial origin of the response keys provide two external frames of reference, an agent-based and a response-based frame, respectively. If participants perform the task with uncrossed (right) hands, the two reference frames are fully aligned and therefore confounded. However, when both actors' hands are crossed, with the left sitting person operating the right response key and vice versa, agent-based and response-based coordinates are misaligned and can thus be deconfounded.

Using this rationale, Welsh (2009) found a visual JSE for uncrossed and crossed hand postures irrespective of whether the participants performed the task with the inner (i.e., right hand of the left actor and left hand of the right co-actor) or the outer hands (i.e., left hand of the left actor and right hand of the right co-actor). This result suggests that the JSE is neither dependent on the spatial origin of the responding agents (i.e., external, agent-based coordinates), nor on the anatomical origin of the responding hands (i.e., internal, anatomical coordinates), but rather tied to the spatial location of the response keys (i.e., external, response-based coordinates; Welsh, 2009; but see Dolk et al., 2013b; Liepelt et al., 2013, for evidence suggesting some flexibility in coding). Further support highlighting the significance of spatial features in representing alternative action events in joint Simon tasks comes from the so-called *transfer-of-learning* paradigm, in which participants perform a number of spatially incompatible responses to left and right stimuli before performing the Simon task (i.e., agents on the right respond to stimuli on the left, whereas the agents on the left respond to stimuli on the right; Milanese et al., 2010, 2011; Ferraro et al., 2012). Practicing spatially incompatible responses have been found to reverse the JSE, irrespective of whether co-actors remain the same or change between practice and joint Simon task (Milanese et al., 2011). However, when co-acting individuals changed their seats in between spatially incompatible practice and subsequent joint Simon task, a normal JSE was observed (Milanese et al., 2011; Ferraro et al., 2012), suggesting that participants do not really represent the social identity of co-actor or action but rather the spatial relationship between action alternatives. Since the JSE-polarity flips as a consequence of changing the spatial relations between agents, responses, and stimuli but not when changing the social identity of co-acting agents, one might argue that the underlying cognitive representation is more reliably fed by and thus, more sensitive to its constituting spatial features than to its social features.

Despite the significance of spatial properties, other studies investigated the impact of more conceptual factors. For example, in a study of Tsai et al. (2008), participants thought they were performing a joint Simon task together with an unseen person (biological agent condition) or a computer (non-biological agent condition), while they were actually interacting with a computer program in both conditions. A JSE only occurred in the biological agent condition, indicating that the belief of interacting with an intentional agent can influence the representation of alternative action events (but see Welsh et al., 2007, for evidence against this view). Recently, Sellaro et al. (2013) showed that the belief to interact with an intentional agent alone is not sufficient to induce the JSE, but that this belief has to be attached to a salient spatial event that occurs next to the participant. Interestingly, once such an alternative salient spatial event is established, ongoing sensory feedback is not needed to keep up the cognitive representation thereof and thus for establishing a JSE (Sebanz et al., 2003; Vlainic et al., 2010).

Further studies investigated the influence of interpersonal relationships on the JSE. Human interactions are, by default, perceived to imply positive interdependence, which motivates people to engage in acts of cooperation (Poortvliet and Darnon, 2010). However, there are situations in daily life, such as competitive contexts, where considering other agents too much may be of disadvantage. Results from the JSE indeed seem to support this assumption. While positive mood or a positive relationship with the co-actor elicited a JSE, bad mood (Kuhbandner et al., 2010), intimidating co-actors (Hommel et al., 2009), or actual competition (i.e., instructions to out-perform others; Iani et al., 2011) abolished or drastically decreased the JSE (but see Ruys and Aarts, 2010, for a more complex picture). These findings show that social factors do have some impact on how people represent their own action vis-à-vis those of others. This conclusion is also supported by the observation that the JSE is increased in members of a collectivistic religion (Colzato et al., 2012a) and in individuals that were primed to attend to the social interdependence of their self (Colzato et al., 2012b) or a divergent style of thinking (Colzato et al., 2013).

Another social factor that affects the JSE is the perceived or real similarity between agent and co-agent. For instance, Müller et al. (2011a) observed that the JSE is more pronounced if co-acting with another (videotaped) human than with a (videotaped) Pinocchio, suggesting that the JSE increases with greater similarity between co-actors. Interestingly, however, the decrease of the effect with dissimilar co-actors can be reduced or eliminated by pre-instructing participants to take the perspective of the non-biological co-actor (e.g., Pinocchio; Müller et al., 2011a) or dissimilar human co-actor (e.g., out-group members; Müller et al., 2011b). Similar findings were also observed for human-robot interaction. When two groups of participants were interacting with the same humanoid robot (controlled by a computer program), the mere pre-instruction of interacting with an “intentional” vs. unintentional robot moderated the degree of action-event-representations, leading to a JSE in the intentional but not in the unintentional condition (Stenzel et al., 2012). These findings show that the JSE is sensitive to perceived interpersonal similarity, which can be increased by priming **anthropomorphic** interpretations (cf., Epley et al., 2007) of actually dissimilar agents.

KEY CONCEPT 4. Anthropomorphism Is the technical term of ascribing human or human-like characteristics, emotion, forms, intentions, motives and many more to animals, events, forces of nature and other things or objects.

#### Electrophysiological and neuroimaging findings

Given that the standard Simon effect is commonly assumed to reflect increased response conflict in S-R-incompatible trials (Kornblum et al., 1990), electrophysiological and neuroimaging techniques have been employed to study response-selection processes and response conflict also in joint Simon tasks. Analyses of event-related potentials (ERPs) showed a larger NoGo-P3, a potential that is associated with action control and response inhibition (e.g., Falkenstein et al., 1995; Bokura et al., 2001), for incompatible nogo trials in joint conditions as compared to solo go-nogo or passive co-actor conditions (Sebanz et al., 2006a; Tsai et al., 2006). This finding has been taken to indicate that more inhibitory control was needed on nogo trials in the joint condition, because representations of pre-instructed alternative action events had to be suppressed to fulfill the joint task requirements (Sebanz et al., 2006a; Tsai et al., 2006; Cavallo et al., 2014). Additionally, Tsai et al. (2006) analyzed the Lateralized Readiness Potential (LRP, time-locked to the stimulus onset), an ERP-component assumed to reflect the stimulus-driven preparation of a manual response (i.e., response selection; Coles, 1989). LRPs on compatible nogo trials and incompatible go trials were also significantly larger in the joint as compared to the solo go-nogo condition, which was taken to indicate priming effects of cortical responses, corresponding to the co-actor's actions (Tsai et al., 2006). Similar results were obtained when Tsai et al. (2008) manipulated the intentionality of the co-actor (see Behavioral Findings).

An fMRI study found stronger activations in medial frontal cortex (MFC) and premotor cortex when participants performed the Simon task together with an active as compared to a passive co-actor, who just rested his/her finger on the alternative response button (Sebanz et al., 2007). This result is in good accordance with other neuroimaging findings highlighting the involvement of MFC, temporoparietal junction (TPJ), superior temporal sulcus (STS), and the temporal poles in social cognition (Adolphs, 2003; Amodio and Frith, 2006; Frith and Frith, 2007). While TPJ, STS, and the temporal poles are typically associated with reasoning about mental states of self and other (e.g., Liepelt et al., 2008b; Spengler et al., 2009; Van Overwalle, 2009), the MFC has been suggested to be involved in monitoring and coding one's own and others' actions (e.g., Frith and Frith, 2007; Radke et al., 2011; Dolk et al., 2012).

#### Patient studies

Although the JSE has been shown to be modulated by the perceived intentionality (Tsai and Brass, 2007; Müller et al., 2011a; Stenzel et al., 2012) or perceived agency (Stenzel et al., 2014) of co-acting agents, these findings do not directly indicate an involvement of social reasoning and high-level social cognitive processes. To investigate whether the effect involves, or is related to the attribution of mental states to others (the so-called Theory-of-Mind, ToM; see Premack and Woodruff, 1978), Sebanz et al. (2005b) conducted a study with high-functioning autistic patients. However, although individuals with autism are generally assumed to have deficits in processing social information (Frith, 2001; Frith and Frith, 2010), there was no evidence that autistic individuals performed the joint Simon task any differently than non-autistic controls.

It is important to emphasize that Sebanz et al. (2005b) studied autistic individuals that either passed first or second-order ToM-tasks, so that they were able to infer another person's mental state in principle (first order) and to infer one person's beliefs about another person's beliefs (second order; Sebanz et al., 2005b). These abilities are considered to reflect residual social processing capacities (Humphreys and Bedford, 2011) that can potentially account for the observed, basically normal JSE. To test for this possibility, Humphreys and Bedford (2011) compared patients with severe lesions in the frontal lobe with patients having lesions in posterior parietal cortex (PPC) and TPJ. The latter failed both first- and second-order ToM-tasks. Explicitly instructing both patient groups to take the other persons' action into account revealed consistent JSEs in patients with temporoparietal lesions, whereas the JSE decreased over time in patients with frontal lesions. Humphreys and Bedford argued that patients with frontal lesions may have difficulties in preserving sufficient processing resources to maintain the other persons' actions on top of coding one's own action events.

In conclusion, since both lesion groups of Humphreys and Bedford failed in ToM-tasks, the available evidence does not provide clear support for the idea that the JSE relies on a particular social mechanism, interpersonal perception or cognition, or high-level social representation (e.g., mental state attribution).

## How social is the joint Simon effect?

The fact that the presence of another active individual is able to increase response conflict, as evidenced by the emergence of the JSE, indicates that agents must consider this presence in one way or the other. The theoretical challenge is to determine what the critical factor is and how it affects the representation of tasks and actions. Several authors have suggested that the agent may automatically co-represent the co-actors task-share, i.e., the “rule that states the stimulus conditions under which a co-actor should perform a certain action” (Sebanz et al., 2005a; p. 1235). According to this *action co-representation* account, co-representation increases the amount of conflict during action selection, which in turn produces the JSE (cf., Sebanz et al., 2003, 2005a, 2006b, 2007; Tsai and Brass, 2007; Vesper et al., 2010; Müller et al., 2011a,b).

Others have argued that the JSE may not reflect the co-representation of the co-actor's S-R rule but, rather, the co-representation of the co-actor him/herself (Wenke et al., 2011). This *actor co-representation* account shifts the focus from the co-actors' mere S-R-based activity to their responsibility to act, and it suggests that the action selection conflict does not reflect difficulties with action-event-discrimination (i.e., with respect to which particular action is to be performed) but with self-other discrimination (i.e., with respect to which particular agent is responsible for an action to be performed in a certain moment in time). In other words, the JSE might reflect uncertainty as to whose turn it is to execute the upcoming action (for a similar line of arguments see, the *agent identification account*; Philipp and Prinz, 2010).

Unfortunately, both of these social approaches fail to explain a number of observations. For one, both would suggest that individual difficulties to represent other people and their actions should reduce or eliminate the JSE, which is inconsistent with the available findings in patients suffering from such difficulties (see Patient Studies). For another, both approaches suggest that the co-actor must actually be an intentional agent or at least be interpreted in anthropomorphic ways. That this is unnecessary was demonstrated by a recent study of Dolk et al. (2011). In several experiments, these authors systematically de-socialized the “joint” Simon task context. First, they showed that a significant “JSE” can be obtained if the actor performs a solo go-nogo Simon task side-by-side a passive observer of the alternative response button that is associated with an attention-attracting event. Next, they demonstrated that the same effect is obtained when the passive observer is absent, suggesting that it was only the attention-attracting event that created the response conflict.

The Dolk et al. (2011) study suggests that neither the integration of another person nor the integration of another person's action into one's own action, task, or body representation is necessary for the JSE to occur. As even non-social events are sufficient to reliably influence an individual's own task performance, it seems to be the presence or expectation of salient events as such that underlies the JSE. Hence, the JSE may be socially induced by the presence of a responding co-actor without necessarily being social in nature. Indeed, Dolk et al. (2013a) observed significant JSEs induced by a Japanese waving cat and a ticking metronome.

As an alternative to the more “social” accounts, Guagnano et al. (2010) suggested that the co-actor's action may serve no other purpose than providing a spatial reference frame. According to this *spatial response coding* account, an actor's own action is coded in relation to the other's action—just like in the standard Simon task, where one's own left-hand action provides a spatial reference for the relative coding of one's right-hand action, and vice versa (Hommel, 1996). Based on their results (which showed a JSE with close but not with distant co-actors), Guagnano et al. (2010) further proposed that such a reference frame is effective only if the co-actor is responding in the participant's peripersonal space (i.e., within arm reach), but not if he or she is responding in the participant's extrapersonal space (i.e., outside of reaching distance; but, see Welsh et al., 2013 for a more complex picture). Note that neither the action or actor co-representation account nor the agent identification account can explain this distance effect, which provides some indirect support for the spatial response coding account. Likewise, the spatial response coding account does not require any social attribution processes, so that it can easily deal with the demonstration of reliable JSEs in patient populations in which processes are impaired (see Patient Studies). At the same time, however, the account fails to explain why JSEs should depend on the agent's mood (Kuhbandner et al., 2010), religious attitude (Colzato et al., 2012a), self-construal (Colzato et al., 2012b), style of thinking (Colzato et al., 2013) or on the personal relationship between actor and co-actor (Hommel et al., 2009).

## A referential coding account

Given the difficulties that social accounts of the JSE have with the effectivity of non-social factors and the opposite problems of the spatial response coding account with explaining the impact of some social factors, Dolk et al. (2013a) suggested a more comprehensive *referential coding* account. In the following, we will introduce the basic assumptions of the referential coding account and illustrate how this account may be able to integrate the available evidence on the JSE.

Performing a (joint or solo) Simon task requires the preparation and selection of intentional actions. According to ideomotor theories of action control (Prinz, 1987; Hommel et al., 2001; Hommel, 2010), actions are represented by codes of their sensory consequences. In particular, TEC (Hommel et al., 2001) assumes that cognitive action representations consist of networks of codes representing the features of all perceivable effects, such as the seen, heard or felt location, direction and speed of an action, the effector it involves, the object it may relate to, and so forth (Hommel, 1997). Action control operates on these perceptual representations and action selection consists in activating the codes of the to-be-generated action effects (i.e., of the perceptual consequences of the action).

**Figure 1:**
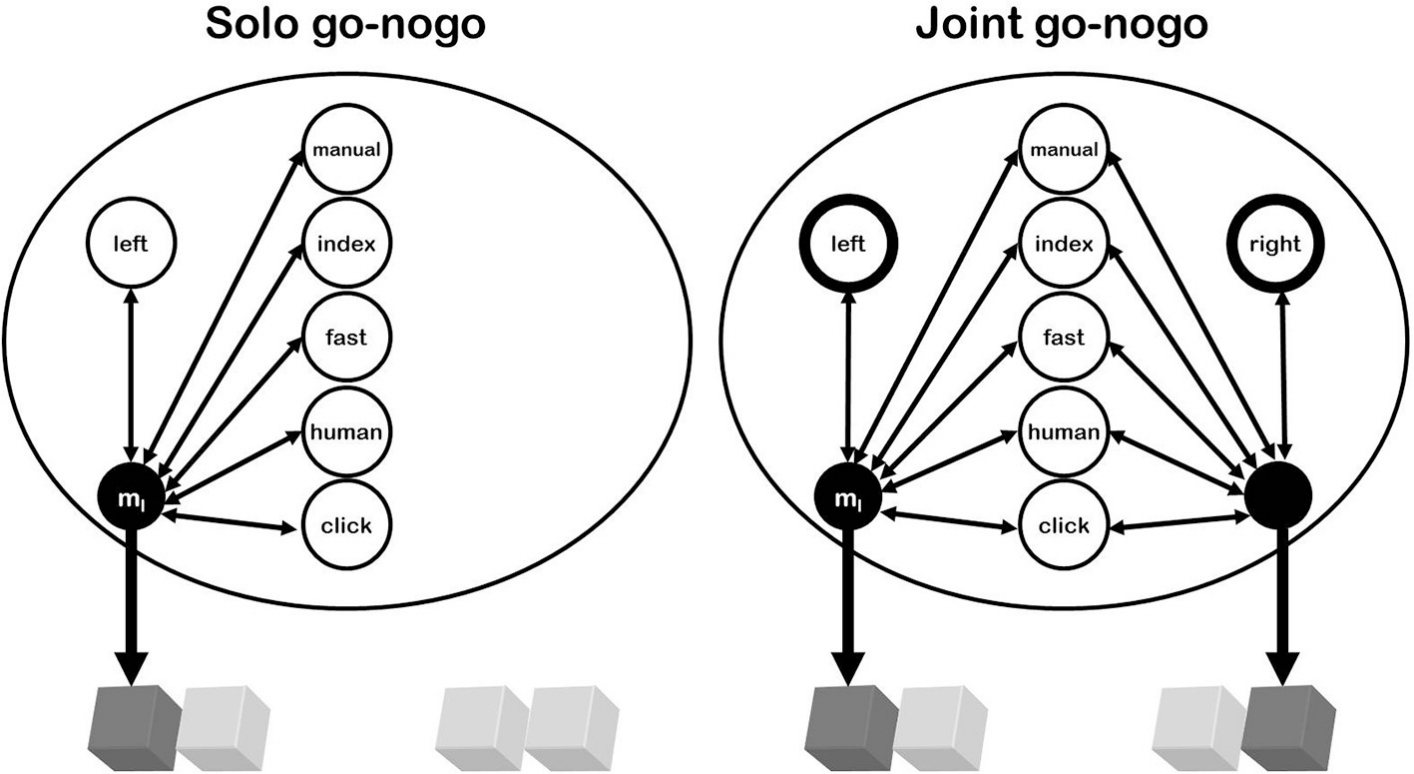
**Referential coding in the go-nogo versions of the Simon task**. The figure shows tasks in which the agent operates the left response key. In the Solo version, a left keypress produces numerous action effects (i.e., something manual and fast, with an index finger moving, something human on the left that comes with a clicking sound, etc.) and can thus be coded in many ways; i.e., be represented by any of these and other action effects. In the Joint condition, the same action effects are produced and could be used for referential coding, but most of them are shared by the other, alternative event. With one exception: the location. Discriminating between the two action events thus requires emphasizing (attending to, weighting more strongly) the corresponding (response) location. This makes the left keypress to be represented mainly as “left.” Any event sharing that feature (such as a target stimulus on the left side) will thus activate the corresponding action more strongly in the Joint than in the Solo task-the Joint Simon effect.

Importantly for our purposes, this rationale of representation implies that one's own actions and the actions of another person are basically represented in the same way (i.e., by means of the same kinds of codes; Hommel, 2009, 2011; see Figure 1). If we assume that response conflict reflects the concurrent activation of more than one action representation (e.g., because of endogenous preparation, stimulus-induced activation, and/or cross talk), this means that actively representing another person's action can create the same kind of response conflict than actively representing more than one of one's own possible actions. In other words, what matters for response conflict is the number of concurrently active action representations but not the source of the activation.

What the actor eventually needs to do is to select the task-relevant action representation from all concurrently activated representations. Concurrent activation thus creates a discrimination problem, which requires a strong focus on those features that discriminate best between task-relevant and task-irrelevant representations (see Ansorge and Wühr, 2004 for a response discrimination account of the standard Simon effect). In the classical Simon task, the most obvious discriminating feature is horizontal location. As a consequence, people are likely to code their responses as “left” and “right,” which equips the active action representations with spatial codes that can interact with equivalent spatial stimulus codes—which in turn creates a *SE* (Hommel et al., 2001). Rendering the task into a go-nogo task by having participants to respond to only one of the two stimuli by performing just one of the two responses eliminates the response-discrimination problem, as now there is just one response being observed and to be executed. Accordingly, there is no need to code responses as “left” or “right” anymore, which explains why the SE typically disappears (Hommel, 1996).

However, as soon as an alternative action is made available, as in the social or non-social go-nogo-Simon task, the discrimination problem is back. Given the typically arranged (joint) workspace (i.e., horizontally or vertically; e.g., Dittrich et al., 2013), it appears reasonable that participants will again emphasize the discriminating features of their responses (i.e., through an increased “intentional weighting” of spatial features; Hommel et al., 2001; Yamaguchi and Proctor, 2012; Memelink and Hommel, 2013; see Figure 1) and thereby code their responses as “left” or “right.” Thus, quite similar to the standard Simon task, an alternative response provides the most obvious reference frame, an assumption we share with the response coding account (e.g., Guagnano et al., 2010). As one would expect from this consideration, no JSE is obtained if participants always react together, so that the two responses need not be discriminated (Lam and Chua, 2009), or if the left-right arrangement of the responses does not match the (vertical) arrangement of the stimuli (Dittrich et al., 2012, 2013).

As TEC does not distinguish between merely perceived events and self-generated events (i.e., perceptions and actions), or between social and non-social events (i.e., living beings and objects), the referential coding account can easily accommodate the observation that non-social events can induce a JSE (Dolk et al., 2011, 2013a). In fact, any representation can create conflict with a representation of the currently (most) relevant response if it is sufficiently active. This implies that the represented event would need to be attended and/or sufficiently salient, which obviously applies to a Japanese waving cat and a ticking metronome, and thus accounts for the observation that non-social events can induce JSEs much like the presence of another human does (Dolk et al., 2013a), but less so to a distant co-actor (Guagnano et al., 2010). Since the referential coding account does not only account for the ability of these and other non-social events to elicit a JSE (see also Tsai et al., 2011; Dittrich et al., 2012), it also explains why the effect decreases with decreasing similarity between perceived and to-be-executed action events (Dolk et al., 2011, 2013a): the more similar two given (action) event representations (anticipated or perceived effects) are the stronger is the cross talk between them. In other words, representations of similar action events are more difficult to discriminate, which makes it more likely that the most obvious and parsimonious event-feature: *spatial location* is used to help with the discrimination. This rationale also accounts for the more pronounced JSEs in human-human interactions compared to interactions with a computer (Tsai et al., 2008), a machine-like robot (Stenzel et al., 2012), or a puppet (Tsai and Brass, 2007): action events produced by non-human, inanimate entities simply exhibit a lesser degree of (perceptual and/or conceptual) similarity with human actions (see Figure 1). It also explains why robot- or puppet-induced JSEs increase in size if these “co-actors” are made to behave intentionally (Stenzel et al., 2012) or otherwise more human-like (Müller et al., 2011a).

As we have discussed above, a number of social factors have been shown to affect the JSE. How does referential coding account for these observations? According to TEC's cognitive binding principle (Hommel, 2004), cognitive representations integrate concurrently active feature codes. It is this principle that accounts for the integration of motor patterns with codes of their perceptual consequences—the key assumption of ideomotor theory (Hommel, 2009). While codes related to immediate action consequences are likely to be activated by performance of the given action, these codes will not be the only ones being active at that time. People can clearly distinguish between their own actions and those carried out by others (even if not all representational systems reflect that difference), which means that codes representing themselves (their body, affective state, goals, etc.) are integrated to some degree with action representations, which provides additional means to discriminate between self-generated and other-generated actions. Again, the self-related codes can overlap with other-related codes to various degrees, depending on the perceived similarity between me and other (see Figure 1). According to the rationale explained above, more similarity would make the discrimination more difficult, which in turn would require more emphasis on the discriminating spatial features. As this emphasis should increase the JSE, this would imply that greater perceived similarity between actor and co-actor should increase the JSE. Note that while the application of this reasoning to the JSE refers to the coding of location (the only obvious candidate in the standard JSE task), any other feature can serve this function, too, as long as it enables sufficient discrimination between (stimulus- and/or action) event-alternatives, and thus provides a reference for coding one's own actions (see, Sellaro et al., under review, for a feature other than location, i.e., color).

As we have discussed already, various observations have confirmed this prediction. Given that interpersonal relationships (e.g., Mikulincer et al., 1998) and group membership (e.g., Aron et al., 1991; Avenanti et al., 2010) have been shown to increase perceived self-other overlap (Davis et al., 1996) and induce a more positive evaluation of the other (Brewer, 1979), it is reasonable to assume that a positive relationship between co-acting individuals or positive mood leads to greater perceived similarity (Heider, 1958). This explains the larger JSEs found under such conditions (Hommel et al., 2009; Kuhbandner et al., 2010), and it accounts for both the disappearance of the JSE when interacting with an out-group member (Müller et al., 2011b) and the reappearance of the effect after instructing participants to take the out-group member's perspective (Müller et al., 2011b).

Taken together, the principle of the referential coding account can be generalized to the following assumption: Self- and other-generated events are cognitively represented by means of codes describing their perceptual features and the perceivable effects they create (e.g., attitude, color, direction, emotions, location, orientation, shape, speed and any other personal and non-personal characteristics; Dolk et al., 2011; Colzato et al., 2012a,b; Hommel, 2013). The degree of similarity would then not necessarily be a qualitative, but rather a gradual one (Hommel, 2013). Accordingly, increasing the degree of similarity increases the demand of discriminating alternative **event-representations**, leading to larger JSEs. This is an interesting methodological implication: The size of the JSE might be taken as an indicator of the similarity between (self- and other-generated) alternative events, and as a measure of the degree of self-other integration, particularly in social contexts—which in turn might make the go-nogo Simon paradigm a valuable educational or rehabilitative tool (Humphreys and Bedford, 2011; Liepelt et al., 2012).

KEY CONCEPT 5. Event-representations According to the Theory of Event Coding (TEC) events (self- and/or other-generated) are cognitively represented by means of codes describing their perceptual features and the perceivable effects they create (e.g., attitude, belief, color, desire, emotion, orientation, shape, sound, speed, and any other characteristics).

### Conflict of interest statement

The authors declare that the research was conducted in the absence of any commercial or financial relationships that could be construed as a potential conflict of interest.
